# Association of preoperative co-occurring intervertebral disc-related degenerative features with one-year lumbar discectomy outcomes: A proposal for and preliminary testing of a novel MRI-based criterion

**DOI:** 10.1016/j.ejro.2026.100729

**Published:** 2026-01-17

**Authors:** Tero Korhonen, Jyri Järvinen, Juha Pesälä, Marianne Haapea, Pietari Kinnunen, Jaakko Niinimäki

**Affiliations:** a Medical Research Center Oulu, Oulu University Hospital and University of Oulu, PO Box 10, Oulu 90029 OYS, Finland; b Department of Diagnostic Radiology, Oulu University Hospital, PO Box 10, Oulu 90029 OYS, Finland; c Department of Orthopedics and Traumatology, Oulu University Hospital, PO Box 10, Oulu 90029 OYS, Finland; d Research Service Unit, Oulu University Hospital, PO Box 10, Oulu 90029 OYS, Finland

**Keywords:** Lumbar, Discectomy, Outcome, Modic changes, Endplate damage, Intervertebral disc degeneration

## Abstract

**Purpose:**

This study developed a criterion for preoperative co-occurring intervertebral disc (IVD)-related degenerative features and evaluated its association with one-year outcomes following single-level lumbar discectomy.

**Methods:**

The novel literature-based criterion, termed “Advanced Preoperative Degeneration” (APD), required the operated segment to exhibit preoperatively at least two advanced-level phenotypes from endplate damage (EPD), Modic changes (MC), and IVD degeneration. Subsequently, a retrospective single-center register-based study of patients treated with single-level micro- or endoscopic lumbar discectomy at a tertiary-level hospital between 2017 and 2022 was performed. The patients were categorized into three groups, APD-positive, APD1/3, and APD0, based on the presence of two or more, one, or none of the required phenotypes, respectively. A mixed-effects model was employed to assess between-group differences in improvement of LBP and leg pain (0–100 VAS), disability (ODI), and quality of life (EQ-5D-3L) from baseline to the one-year postoperative time point.

**Results:**

The cohort consisted of 140 patients (mean age: 45.3 years; 81 [57.9 %] male). Overall, the patients exhibited significant improvements in all PROMs after discectomy. However, at the one-year follow-up, the APD-positive group exhibited significantly higher leg pain and disability levels than the APD0 group, with mean scores of 31.4 versus 19.6 for leg pain and 20.6 versus 12.0 for ODI, respectively.

**Conclusion:**

This study introduces a novel approach by integrating preoperative co-occurring IVD-related degenerative features into a composite APD criterion. Meeting the APD criterion was associated with significantly poorer one-year outcomes for leg pain and disability following lumbar discectomy.

## 1 Introduction

Radiculopathy, characterized by pain following the dermatomal path of a lumbar nerve root, exhibits an annual prevalence of approximately 10–25 %. Lumbar disc herniation (LDH) stands as a principal etiology of radiculopathy, with discectomy being a standard operative intervention [1], [2]. Nonetheless, the persistence of low back pain (LBP) or leg pain after discectomy remains notable. A secondary analysis of the SPORT study revealed 29 % and 20 % cumulative risks of LBP and leg pain recurrences, respectively, within one year [3]. Meanwhile, a systematic review concluded that recurrent LBP or leg pain affects 5–36 % of patients two years postoperatively [4]. Progressive degenerative changes in the operated lumbar segment are implicated in the etiology of post-discectomy pain. Longitudinal radiological data over one to five years post-discectomy indicate progressive damage to endplates, the formation of Modic changes (MC), and intervertebral disc (IVD) height loss, with some studies associating such progression with residual pain [5], [6], [7].

Postoperative degenerative changes’ importance to post-discectomy LBP and leg pain may be explained by the anatomical failure mechanisms associated with LDH. Radiological and histopathological findings indicate that approximately 50 % of LDHs involve damage to the endplate, a composite cartilaginous and osseous barrier that delineates the IVD from adjacent vertebrae [8], [9]. The endplate is crucial for biomechanical stability and nutrient supply to the IVD, and endplate damage (EPD) can initiate segmental degeneration [10]. Consequently, endplate-induced IVD degeneration is recognized as a specific degenerative subtype [11]. Moreover, EPD has been identified as a predisposing factor for segmental degeneration progression, involving MC activity and IVD degeneration advancement [12].

Hence, lumbar discectomy outcomes may be influenced by the level of preoperative degeneration in the operated segment. Despite the demonstrated clinical significance of coexisting degenerative features for LBP severity [13], [14], no study has evaluated the preoperative impact of co-occurring degenerative features on patient-reported outcome measures (PROMs) for lumbar discectomy. This study proposes a novel literature-based criterion termed “Advanced Preoperative Degeneration” (APD) for assessing IVD-related degenerative features’ preoperative co-occurrence. This study also preliminarily tests this criterion’s association with one-year discectomy outcomes.

## 2 Materials and methods

### 2.1 Development of the criterion

**Fig. 1:**
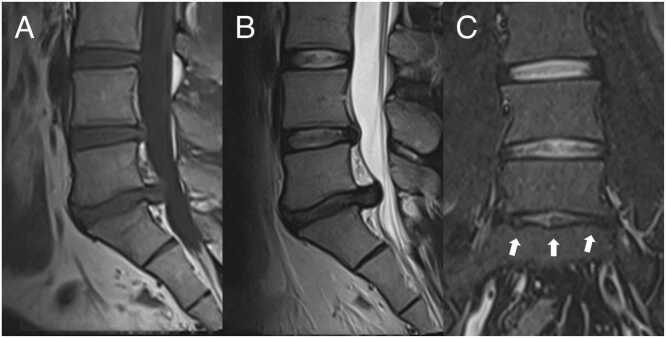
A 34-year-old patient with lumbar disc herniation at L5/S1 shown in preoperative lumbar MRI: sagittal T1-weighted (a), sagittal T2-weighted (b), and coronal STIR (c) sequences. The operated segment demonstrated a Pfirrmann grade 4 intervertebral disc. Modic type 2 changes were present in both sides of the segment. The cranial endplate showed < 25 % damage, whereas the caudal endplate exhibited ≥ 25 % damage (arrows). The combination of Pfirrmann grade ≥ 4 and ≥ 25 % endplate damage fulfilled the APD criterion.

To evaluate the co-occurrence of advanced-level IVD-related degenerative phenotypes before lumbar discectomy, the current authors, including senior musculoskeletal radiologists and spine surgeons, collaboratively developed the APD criterion. This criterion, derived from evidence in earlier lumbar degeneration studies, was based entirely on common MRI findings to enhance clinical and academic utility. The APD criterion comprises three advanced-level degenerative phenotypes from EPD, MC, and IVD degeneration, which are detailed below and in Table 1. The criterion is met with the preoperative co-occurrence of at least two of these phenotypes in the operated lumbar segment, as shown by the example in Fig. 1. Given late-stage IVD degeneration’s potential protective effect against LDH [15], requiring the presence of all three phenotypes would diminish the criterion’s clinical utility. Therefore, this requirement was rejected.

**Table 1:** The MRI-based criterion for advanced preoperative degeneration (APD).

Feature	Criterion
EPD	Area of damage ≥ 25 %
MC	MC type I: pure or predominancy in mixed MC lesions
IVD degeneration	Pfirrmann grade ≥ 4
The criterion for APD is met if ≥ 2 of the required phenotypes are preoperatively present in the operated lumbar segment.^a^	

EPD, endplate damage; MC, Modic changes; IVD, intervertebral disc; APD, advanced preoperative degeneration.

a If met based on EPD and MC, the phenotypes had to be on the same side of the segment.

#### 2.1.1 Endplate damage

The threshold for EPD was established at an area of damage ≥ 25 % relative to the affected endplate, corresponding to a grade ≥ 5 in the Endplate Score (EPS) classification [16]. Recent findings by Zehra et al. [17] demonstrated that a width-based EPD–endplate ratio of 0.20–0.32 serves as a distinct cutoff for various degenerative features, including MC and IVD degeneration. Moreover, EPD size is positively associated with MC’s presence and higher IVD degeneration grades, and importantly, an EPS cutoff ≥ 4 has been linked to the progression of MC and IVD degeneration [12]. Additionally, a sum score ≥ 6 for the EPSs of the cranial and caudal endplates indicates a significantly higher likelihood of MC’s presence and advanced IVD degeneration [16], [18].

#### 2.1.2 Modic changes

MC type 1 (MC1) was included in the criterion due to its role as the most active subtype and its suggested correlation with the adjacent IVD’s accelerated degeneration [19], [20]. Of the subtypes, MC1 exhibits the strongest association with LBP, and correspondingly MC1’s regression is associated with LBP relief [21], [22]. Since MC lesions can comprise mixed subtypes and may be converted from one type to another [21], [22], only pure or predominant MC1 were considered to avoid the inclusion of potential regressing and less clinically significant lesions. If the APD criterion was fulfilled based on EPD and MC phenotypes, these phenotypes had to be located on the same side of the segment, given the significant correlation observed between the EPD’s extent and MC’s presence at the level of an individual endplate–bone marrow complex [12].

#### 2.1.3 Intervertebral disc degeneration

A Pfirrmann grade ≥ 4 for IVD degeneration was chosen because it signifies such macroscopic degenerative changes that have led to at least a moderately collapsed IVD height [23]. This threshold is consistent with previous studies that have regarded a grade ≥ 4 as indicative of a degenerated IVD [12], [16]. Furthermore, Jamaludin et al. [24] demonstrated the strongest association between Pfirrmann grade ≥ 4 and LBP for the two most caudal lumbar segments among patients under 50 years old—demographic characteristics seen in the typical population of patients undergoing lumbar discectomy [25].

### 2.2 Sensitivity analyses

Two additional criteria were included in sensitivity analyses. The first analysis included the size of the MC, while the second criterion, termed the “Modic–endplate complex” (MEC), was adapted from a previous cervical spine study by Baker et al. [26]. An overview of these criteria is provided in sections 1.1 and 1.2 of the Supplemental material.

### 2.3 Clinical testing of criteria

The preoperative values of the novel APD criterion and the two additional criteria for one-year discectomy outcomes were tested retrospectively with a cohort of lumbar discectomy patients from a single tertiary-level hospital.

#### 2.3.1 Patient population

From the Finnish spine register (FinSpine), prospectively collected data from patients who had undergone single-level lumbar discectomy for LDH with radiculopathy and corresponding MRI findings at a single tertiary-level center between October 2017 and September 2022 were included [27]. The exclusion criteria included prior lumbar surgery, a preoperative MRI dated more than six months before the surgery date, missing pre- or one-year postoperative PROMs data, and repeat operation during the follow-up period. The urgency of the surgery was not used as a criterion for exclusion.

#### 2.3.2 Demographic and clinical data

Basic demographic and clinical data were extracted from the FinSpine register. These data comprised patients’ age, sex, body mass index (BMI), smoking status, symptom duration, preoperative mental health status, preoperative cauda equina syndrome, preoperative motor deficit of leg that impaired walking (defined as leg strength ≤ 3 out of 5), and surgery level.

The pre- and postoperative PROMs included LBP and leg pain on a 0–100 Visual Analogue Scale (VAS), disability measured according to the Oswestry Disability Index (ODI), version 2.0 [28] and health-related quality of life (QoL) assessed using the EQ-5D-3L scale [29]. The EQ-5D-3L scale comprises a descriptive system detailing various aspects of QoL and a 0–100 VAS (EQ-VAS) for overall health assessment. The aspects of QoL were converted into index values (EQ-index) ranging from 1.000 (optimal health state) to −0.011, using the Finnish valuation set from the EuroQoL office. In the EQ-VAS, higher scores represent better QoL. Notably, the EQ-VAS slider was oriented horizontally due to the design of the register, contrary to the vertical orientation recommended by the EuroQoL office.

#### 2.3.3 Magnetic resonance imaging and image analysis

All patients underwent a preoperative 1.5- or 3.0-T lumbar MRI with standard spinal imaging protocol, where typical MRI sequences were sagittal T1-weighted (T1w) spin echo (SE) with repetition time (TR) of 552 ms and echo time (TE) of 14.3 ms (or sagittal fluid-attenuated inversion recovery [FLAIR] with TR/TE of 1800/860 ms and inversion time [TI] of 9.4 ms); sagittal T2-weighted (T2w) fast-spin echo (FSE) with TR/TE of 3500/87 ms; sagittal or coronal short-tau inversion recovery (STIR) with TR/TE/TI of 3700/150/66 ms; axial T2w FSE with TR/TE of 4810/97 ms; slice thickness 3–5 mm; and spacing 3.3–6.5 mm.

Two senior musculoskeletal radiologists (JJ and JN), without access to patient information except for the operated lumbar segment, analyzed the MRIs using clinical workstations. The operated lumbar segments were assessed for features included in the APD criterion and the two additional criteria (Table 1 and Tables 7 and 8 in the Supplemental material). JN conducted 63.6 % of the evaluations. To assess intra- and interobserver reliabilities, both radiologists repeated their analysis of 15 randomly selected MRIs, and 19 randomly selected MRIs were analyzed by both of the radiologists, lacking access to the previous findings. These analyses encompassed the entire lumbar spine to increase the required phenotypes’ prevalence.

#### 2.3.4 Statistical analysis

For statistical analysis, the study population was categorized into APD-positive (patients with two or more of the required phenotypes), APD1/3 (patients with one phenotype), and APD0 (patients with no phenotype). Descriptive statistics were presented as means ± standard deviations (SD) or frequencies with percentages. Continuous variables were compared using a one-way analysis of variance (ANOVA), while dichotomous variables were analyzed with chi-square tests. If two different APD grades were present within a segment, patients were categorized according to the highest grade for reporting descriptive statistics. For statistically significant between-group differences, post hoc comparisons were conducted; Tukey’s test was used for the ANOVA, and Holm–Bonferroni-corrected pairwise comparisons were used for the chi-square tests.

A linear mixed-effects model was subsequently fitted to estimate the APD criterion’s association with one-year lumbar discectomy outcomes. The relevant PROMs parameter was used as the dependent variable, with the patient representing the subject factor and the side of the segment serving as a within-subject factor to account for instances where two different APD grades were present within a segment. The model included the interaction term “group * follow-up” and was adjusted for age, sex, BMI, smoking status, symptom duration, preoperative mental health status, and preoperative motor deficit of leg. The same methodology was applied to test the two additional criteria in sensitivity analyses.

Due to low incidence rates, certain parameters were combined for statistical feasibility. Symptom duration was categorized as < 12 weeks, 3–12 months, or > 12 months, and preoperative mental health status was categorized as the presence or absence of anxiety or depression (Question 5 of the EQ-5D-3L: 2–3 or 1, respectively). Cauda equina syndrome occurred exceedingly rarely; therefore, it was excluded from the analysis. Cohen’s kappa (κ) and prevalence-adjusted, bias-adjusted kappa (PABAK) [30] were calculated for intra- and interobserver reliabilities in detecting the phenotypes included in the APD criterion and APD-positivity; this interpretation followed the work of Landis and Koch [31]. Statistical significance was set to a p-value < 0.05. Statistical analyses were performed using IBM SPSS, version 29 (IBM Corp., Armonk, NY, USA).

## 3 Results

### 3.1 Demographic and clinical characteristics

From the 593 lumbar discectomy patients identified in the register, 140 (23.6 %; mean age: 45.3 ± 14.0 years; 81 [57.9 %] male) were included after the remainder were excluded for unanswered pre- or one-year postoperative PROMs (n = 429), previous lumbar surgery (n = 14), an MRI more than six months before surgery (n = 5), and a repeat operation during the follow-up period (n = 5). Microdiscectomy was performed for 136 (97.1 %) patients, while endoscopic discectomy was performed for four (2.9 %) patients. The demographic and clinical characteristics are summarized in Table 2.

**Table 2:** The demographic and clinical baseline characteristics of the study sample.

	Total	APD0	APD1/3	APD-positive	p
N (%)	140 (100.0 %)	29 (20.7 %)	66 (47.1 %)	45 (32.1 %)	
Age, mean (SD)	45.3 (14.0)	43.6 (13.5)	44.6 (13.9)	47.5 (14.6)	0.431
Sex					0.081
Male, N (%)	81 (57.9 %)	22 (75.9 %)	36 (54.5 %)	23 (51.1 %)	
Female, N (%)	59 (42.1 %)	7 (24.1 %)	30 (45.5 %)	22 (48.9 %)	
BMI, mean (SD)	28.0 (5.5)	28.1 (4.8)	27.9 (5.7)	28.2 (5.8)	0.968
Active smoking					0.567
Yes, N (%)	27 (19.3 %)	4 (13.8 %)	15 (22.7 %)	8 (17.8 %)	
No, N (%)	110 (78.6 %)	25 (86.2 %)	49 (74.2 %)	36 (80.0 %)	
Missing data, N (%)	3 (2.1 %)	-	2 (3.0 %)	1 (2.2 %)	
Preoperative anxiety					0.098
Yes, N (%)	39 (27.9 %)	4 (13.8 %)	23 (34.8 %)	12 (26.7 %)	
No, N (%)	99 (70.7 %)	25 (86.2 %)	41 (62.1 %)	33 (73.3 %)	
Missing data, N (%)	2 (1.4 %)	-	2 (3.0 %)	-	
Duration of symptoms					**0.007**
< 6 weeks, N (%)	22 (15.7 %)	6 (20.7 %)	7 (10.6 %)	9 (20.0 %)	
6–12 weeks, N (%)	28 (20.0 %)	10 (34.5 %)	14 (21.2 %)	4 (8.9 %)	
3–12 months, N (%)	58 (41.4 %)	9 (31.0 %)	34 (51.5 %)	15 (33.3 %)	
> 12 months, N (%)	32 (22.9 %)	4 (13.8 %)	11 (16.7 %)	17 (37.8 %)	
Level of herniation					0.427
L1-L2, N (%)	-	-	-	-	
L2-L3, N (%)	5 (3.6 %)	1 (3.4 %)	2 (3.0 %)	2 (4.4 %)	
L3-L4, N (%)	14 (10.0 %)	4 (13.8 %)	5 (7.6 %)	5 (11.1 %)	
L4-L5, N (%)	66 (47.1 %)	18 (62.1 %)	29 (43.9 %)	19 (42.2 %)	
L5-S1, N (%)	55 (39.3 %)	6 (20.7 %)	30 (45.5 %)	19 (42.2 %)	
Preoperative motor deficit of leg impairing walking					0.414
Yes, N (%)	44 (31.4 %)	12 (41.4 %)	20 (30.3 %)	12 (26.7 %)	
No, N (%)	96 (68.6 %)	17 (58.6 %)	46 (69.7 %)	33 (73.3 %)	
Preoperative cauda equina syndrome					> 0.99
Yes, N (%)	3 (2.1 %)	1 (3.4 %)	1 (1.5 %)	1 (2.2 %)	
No, N (%)	137 (97.9 %)	28 (96.6 %)	65 (98.5 %)	44 (97.8 %)	
Preoperative PROMs assessment-operation interval, mean (SD), days	8.8 (8.4)	9.5 (8.4)	8.6 (8.8)	8.8 (7.8)	0.897
MRI-operation interval, mean (SD), days	44.7 (45.4)	45.2 (46.1)	44.3 (44.5)	44.8 (47.3)	0.996

BMI, body mass index; PROMs, patient-reported outcome measures.

Baseline analysis revealed a significant difference in symptom duration across the groups (p = 0.007), with the APD-positive group exhibiting significantly longer durations than the APD1/3 and APD0 groups (p = 0.011 and p = 0.020, respectively). Additionally, a significant difference in baseline leg pain levels (p = 0.050) was observed. This difference was attributable to higher values in the APD1/3 group compared to the APD-positive group (p = 0.040; Table 3).

**Table 3:** Unadjusted pre- and post-operative patient-reported outcome measures (PROMs) of the total study sample and by group.

	Total	APD0	APD1/3	APD-positive	p^a^
LBP, mean (SD)					
Baseline	52.3 (29.2)	46.0 (28.2)	57.5 (29.7)	48.0 (28.5)	0.146
Follow-up	26.1 (26.2)	24.2 (22.9)	23.6 (23.7)	30.8 (31.1)	0.468
Leg pain, mean (SD)					
Baseline	67.2 (26.7)	66.0 (31.6)	72.8 (24.5)	59.3 (25.4)	**0.050**
Follow-up	27.5 (29.1)	19.6 (23.3)	28.2 (29.0)	31.4 (32.2)	0.389
ODI, mean (SD)					
Baseline	45.4 (16.4)	43.8 (15.1)	47.2 (17.2)	44.0 (16.2)	0.522
Follow-up	16.1 (16.7)	12.0 (11.0)	14.7 (15.6)	20.6 (20.1)	0.127
EQ-index, mean (SD)					
Baseline	0.50 (0.15)	0.54 (0.10)	0.47 (0.17)	0.50 (0.15)	0.090
Follow-up	0.73 (0.20)	0.72 (0.17)	0.74 (0.19)	0.71 (0.23)	0.789
EQ-VAS, mean (SD)					
Baseline	45.8 (22.7)	48.4 (19.5)	42.7 (24.0)	48.6 (22.5)	0.331
Follow-up	72.7 (23.2)	67.7 (23.1)	74.0 (23.4)	73.9 (23.4)	0.561

APD, advanced preoperative degeneration; LBP, low back pain; ODI, Oswestry Disability Index.

a For between-group differences in baseline and follow-up values.

### 3.2 MRI findings

The mean interval between a patient’s MRI and operation was 44.7 ± 45.4 days. Forty-five (32.1 %) patients were APD-positive, 66 (47.1 %) were in the APD1/3 group, and 29 (20.7 %) belonged to the APD0 group. Intra- and interrater reliabilities for detecting the required APD phenotypes and APD-positivity were substantial to almost perfect, except for moderate interrater reliability for the MC phenotype (Table 4).

**Table 4:** Intra- and interobserver reliabilities for the detection of the required phenotypes and APD positivity.

	Intra (reader 1)		Intra (reader 2)		Inter (reader 1&2)	
	Cohen’s kappa(95 % CI)	PABAK (95 % CI)	Cohen’s kappa(95 % CI)	PABAK (95 % CI)	Cohen’s kappa(95 % CI)	PABAK (95 % CI)
EPD	0.66(0.45–0.86)	0.88(0.78–0.94)	0.72(0.48–0.95)	0.93(0.85–0.98)	0.71(0.53–0.90)	0.92(0.83–0.96)
MC	0.94(0.83–1.0)	0.99(0.93–1.00)	0.61(0.35–0.87)	0.91(0.81–0.96)	0.42(0.12–0.71)	0.89(0.81–0.95)
IVD degeneration	1.00(1.00–1.00)	1.00(0.90–1.00)	0.94(0.86–1.00)	0.95(0.81–0.99)	0.91(0.82–1.00)	0.92(0.79–0.98)
APD criterion	0.75(0.59–0.90)	0.88(0.78–0.94)	0.66(0.45–0.86)	0.88(0.78–0.94)	0.69(0.51–0.86)	0.88(0.80–0.94)

PABAK, prevalence-adjusted bias-adjusted kappa; 95 % CI, 95 % confidence interval; EPD, endplate damage; MC, Modic changes; IVD, intervertebral disc; APD, advanced preoperative degeneration.

### 3.3 Outcome analysis

**Fig. 2:**
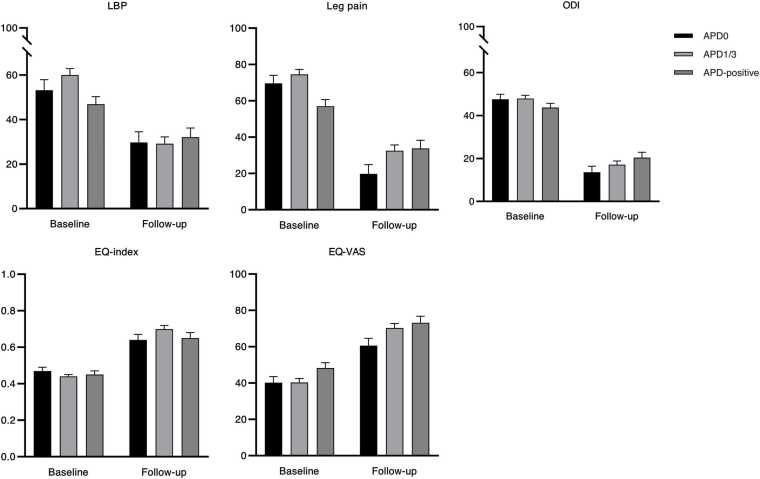
Bar chart with standard errors displaying the estimated means of patient-reported outcome measures (PROMs) for each group.

Significant differences in estimated mean baseline values were observed for leg pain (APD0: 69.6; APD1/3: 74.5; and APD-positive: 57.0; p = 0.012) and EQ-VAS (APD0: 40.2; APD1/3: 40.3; and APD-positive: 48.3; p = 0.007) but not for LBP (p = 0.267), ODI (p = 0.639), or EQ-index (p = 0.588). Table 5 and Fig. 2 display the baseline and follow-up PROMs estimates for each group.

**Table 5:** Estimated means of the groups’ patient-reported outcome measures (PROMs).

	APD0	APD1/3	APD-positive
LBP, est. mean (s.e.)			
Baseline	53.2 (4.7)	60.0 (2.9)	46.9 (3.4)
Follow-up	29.7 (4.8)	29.2 (3.0)	32.1 (4.1)
p^a^	**0.038**		
Leg pain, est. mean (s.e.)			
Baseline	69.6 (4.4)	74.5 (2.8)	57.0 (3.7)
Follow-up	19.8 (5.1)	32.5 (3.2)	33.8 (4.5)
p^a^	**0.002**		
ODI, est. mean (s.e.)			
Baseline	47.6 (2.3)	47.9 (1.5)	43.7 (2.0)
Follow-up	13.6 (2.8)	17.1 (1.7)	20.4 (2.5)
p^a^	**0.034**		
EQ-index, est. mean (s.e.)			
Baseline	0.47 (0.02)	0.44 (0.01)	0.45 (0.02)
Follow-up	0.64 (0.03)	0.70 (0.02)	0.65 (0.03)
p^a^	0.129		
EQ-VAS, est. mean (s.e.)			
Baseline	40.2 (3.3)	40.3 (2.2)	48.3 (2.9)
Follow-up	60.6 (4.0)	70.3 (2.5)	73.2 (3.6)
p^a^	0.199		

Adjusted for age, sex, BMI, smoking status, symptom duration, preoperative mental health status, and preoperative motor deficit of leg.

95 % CIs: estimate ± 1.96 × s.e.

APD, advanced preoperative degeneration; Est. mean, an estimated mean; s.e., a standard error; LBP, low back pain; ODI, Oswestry Disability Index.

a For between-group differences in improvement rates.

All groups exhibited significant improvement in all PROMs. Meanwhile, significant between-group differences were noted in LBP (p = 0.038), leg pain (p = 0.002), and ODI (p = 0.034) but not in QoL estimates (p = 0.129 for EQ-index and p = 0.199 for EQ-VAS) (Table 5).

Compared to the APD0 group, the APD-positive group had statistically significant regression coefficients for leg pain (+26.5 (s.e. 8.1) p = 0.001) and ODI (+10.7 (s.e. 4.4), p = 0.016), which quantify how much smaller the improvement from baseline to one-year follow-up was, on average, in the APD-positive group relative to the APD0 group. Those of the APD1/3 group were not statistically significant for any of the PROMs (Table 6).

**Table 6:** Regression coefficients of the patient-reported outcome measures (PROMs) by group.

	Follow-up			APD1/3			APD-positive		
	B^a^	s.e.	p	B^b^	s.e.	p	B^b^	s.e.	p
LBP	-23.5	6.0	**< 0.001**	-7.3	7.1	0.301	8.8	7.9	0.269
Leg pain	-49.7	6.1	**< 0.001**	7.8	7.1	0.276	26.5	8.1	**0.001**
ODI	-33.9	3.3	**< 0.001**	3.2	3.9	0.416	10.7	4.4	**0.016**
EQ-index	0.18	0.04	**< 0.001**	0.08	0.04	0.073	0.02	0.05	0.666
EQ-VAS	20.4	4.7	**< 0.001**	9.6	5.6	0.085	4.5	6.3	0.477

Adjusted for age, sex, BMI, smoking status, symptom duration, preoperative mental health status, and preoperative motor deficit of leg.

95 % CIs: estimate ± 1.96 × s.e.

APD, advanced preoperative degeneration; B, a regression coefficient; s.e., a standard error; LBP, low back pain; ODI, Oswestry Disability Index.

a For the overall effect of surgery.

b The group * follow-up interaction term reflects the difference in change from baseline to one-year follow-up for each group compared to the APD0 reference group.

### 3.4 Sensitivity analysis

The MC-size-based criterion was associated with significant between-group differences in leg pain (p = 0.028). The regression coefficients for the 1/3 group were statistically significant for leg pain (+18.5, p = 0.008) and ODI (+8.3, p = 0.026). Meanwhile, the regression coefficients for the positive group were not significant for any of the PROMs. The MEC criterion was associated with significant between-group differences in EQ-index (p = 0.032), for which the regression coefficient was statistically significant for the MEC-positive group (−0.069, p = 0.032). Data are presented in Tables 9 and 10 of the Supplemental material.

## 4 Discussion

The primary aim of this study was to evaluate the association between preoperative co-occurring IVD-related degenerative features and one-year outcomes after single-level lumbar discectomy. Based on previous literature, a novel MRI-based criterion for the co-occurrence of the features, termed the “APD criterion”, was established and retrospectively tested on a patient cohort. A significant association was demonstrated between meeting the APD criterion and less improvement in leg pain and disability from baseline to one-year follow-up, compared to the APD0 reference group. The mean unadjusted one-year postoperative leg pain values were 31.4 and 19.6 on a 0–100 VAS, while the ODI values were 20.6 and 12.0 for patients in the APD-positive and APD0 groups, respectively. The difference between these values was also close to the absolute minimal clinically important differences (MCID) of 15 for VAS and 10 for the ODI [32]. The findings of this study contribute to the enhancement of clinical preoperative patient information, especially for the APD-positive patient population. Additionally, we recommend that greater academic and clinical focus be directed toward the management of LDH in APD-positive patients to reduce disparities in treatment outcomes.

A meta-analysis of PROMs’ trajectories after lumbar discectomy revealed that, although leg pain relief is immediate after discectomy, low-grade leg pain may persist in the long term, alongside LBP and disability [33]. Similarly, in the present study, the APD-positive group reported higher levels of postoperative leg pain, with a substantially smaller reduction in leg pain from preoperative levels compared to other groups. The APD-positive group also exhibited significantly longer symptom duration, which is a known risk factor for poorer lumbar discectomy outcomes [34], [35]. However, in the fully adjusted model, including symptom duration, the difference remained significant, suggesting that the APD criterion may indeed contribute to pathological postoperative changes, which could be explained by three primary theories.

First, surgical removal of the LDH may lead to biomechanical alterations in the operated segment, potentially reducing the IVD space height, which is likely somewhat compromised already in the case of APD positivity. Such changes, combined with potential postoperative instability, may increase the likelihood of residual mechanical compression of the affected nerve root in APD-positive patients [7], [36]. Second, biochemical factors may also contribute to the heightened postoperative leg pain. Degenerated IVDs are known to secrete elevated levels of pain-related inflammatory molecules, including tumor necrosis factor (TNF) alpha [37]. Due to the presumed greater degrees of postoperative segmental degeneration, the APD-positive group may exhibit increased secretion of these molecules, resulting in sustained chemical irritation of the nerve root.

Third, APD-positive patients may have an increased susceptibility to residual nerve root irritation due to recurrent LDH (rLDH). In their meta-analysis, Brooks et al. [15] identified preoperative MC1 as a significant risk factor for rLDH, a phenotype included in the APD criterion of the present study. Additionally, they highlighted an elevated disc height index, indicative of preserved IVD space, as another important risk factor for rLDH. Conversely, Pfirrmann grades 1–3 versus 4–5 did not predict rLDH. The present study demonstrated that individual advanced-level phenotypes, such as MC1, did not significantly influence PROMs, as evidenced by the outcomes of the APD1/3 group. These findings suggest that a composite preoperative phenotype of MC1 and advanced EPD may present a greater risk for rLDH compared to MC1 alone, potentially explaining the observations in the APD-positive group.

The current study’s strengths include its detailed characterization of MRI findings, the APD criterion’s robust foundation in the previous literature, and the use of various PROMs. Importantly, however, certain limitations should be recognized. The study’s design was retrospective and observational, and a considerable number of patients had to be excluded due to unanswered PROMs queries. This lack of responses is likely attributable to the novelty of the register in the participating hospital and the ongoing development of its clinical utilization [27]. However, the complete elimination of selection bias cannot be guaranteed; therefore, planning is actively underway for a multi-center validation study of the APD criterion. Moreover, some significant demographic confounders, such as somatic comorbidities and educational level, were not controlled for. Symptoms related to postoperative degenerative changes, which may evolve over time [38], [39], might not have been fully evident within the one-year follow-up period. Moreover, the assessment of MRI features was limited to EPD, MC, and IVD degeneration; consequently, other MRI features—such as facet joint pathology—remained uncontrolled for, and they may have influenced the study’s outcomes.

In conclusion, preoperatively meeting the novel APD criterion, which indicates the presence of two or more advanced-level IVD-related degenerative phenotypes in the operated segment, was significantly associated with less improvement in leg pain and disability at one year after single-level lumbar discectomy.

## CRediT authorship contribution statement

**Pietari Kinnunen:** Writing – review & editing, Methodology, Investigation, Data curation. **Marianne Haapea:** Writing – review & editing, Visualization, Software, Methodology, Formal analysis. **Jaakko Niinimäki:** Writing – review & editing, Validation, Supervision, Project administration, Methodology, Investigation, Conceptualization. **Tero Korhonen:** Writing – review & editing, Writing – original draft, Validation, Methodology, Investigation, Data curation, Conceptualization. **Juha Pesälä:** Writing – review & editing, Resources, Methodology, Investigation, Data curation, Conceptualization. **Jyri Järvinen:** Writing – review & editing, Validation, Methodology, Investigation.

## Consent

Informed consent from patients was not required, as the study was a retrospective database analysis

## Ethics approval

Approval for the study protocol was granted by the regional ethics committee of the hospital. The study group adhered to the principles outlined in the World Medical Association's Helsinki Declaration.

## Funding

No financial or other support by organizations that may gain or lose financially through publication of this manuscript were received.

## Declaration of Competing Interest

The authors declare that they have no known competing financial interests or personal relationships that could have appeared to influence the work reported in this paper.

## Data Availability

The data that support the findings of this study are available from Finnish Institute for Health and Welfare, but restrictions apply to the availability of these data, which were used under license for the current study and so are not publicly available.

## Footnotes

Appendix A Supplementary data associated with this article can be found in the online version at doi:10.1016/j.ejro.2026.100729.
