# Supplementary material for: Association of preoperative co-occurring intervertebral disc-related degenerative features with one-year lumbar discectomy outcomes: A proposal for and preliminary testing of a novel MRI-based criterion
Source: Eur J Radiol Open. 2026 Jan 17;16:100729. doi: 10.1016/j.ejro.2026.100729 (PMC12855554; doi:10.1016/j.ejro.2026.100729)
Supplement: Supplementary file 1 — Supplementary material [file mmc1.docx]

Association of preoperative co-occurring intervertebral disc-related degenerative features with one-year lumbar discectomy outcomes: a proposal for and preliminary testing of a novel MRI-based criterion

Supplemental material for sensitivity analysis

European Journal of Radiology Open

1. Overview and rationale of the criteria

1.1. MC-size-based criterion

Previous literature indicates that the extent of Modic changes (MC) may hold clinical significance, showing a positive correlation with the adjacent intervertebral disc’s (IVD) advanced degeneration [1,2]. Additionally, two large-scale studies have reported a stronger association between more extensive MC and low back pain (LBP) [3,4]. Therefore, the criterion used in the first sensitivity analysis modified the MC phenotype requirement. This modification included either a sagittal peak height or an axial extent of ≥ 50% relative to the affected vertebra without considering the MC type (Table 7).

**Table 7** The MC-size-based criterion included in the sensitivity analysis.

| Feature | Criterion |
| --- | --- |
| EPD | Area of damage ≥ 25% |
| MC | Height ≥50% or extent ≥ 50% |
| IVD degeneration | Pfirrmann grade ≥ 4 |
| The criterion is met if ≥2 of the required phenotypes are present.^1^ | |

^1^ If met based on EPD and MC, the phenotypes had to be on the same side of the segment.

EPD, endplate damage; MC, Modic changes; IVD, intervertebral disc.

1.2. Modic–endplate complex

The second sensitivity analysis was based on the study by Baker et al. [5] in which they introduced the cervical Modic–endplate complex (MEC), requiring the presence of MC and endplate damage (EPD) in a single cervical segment. In acknowledgment of the significant differences between the work of Baker et al. and the present study, the second sensitivity analysis focused on MEC’s preoperative presence in the operated lumbar segment (Table 8).

**Table 8** The MEC criterion included in the sensitivity analysis.

| Feature | Criterion |
| --- | --- |
| EPD | Any EPD |
| MC | Any MC |
| The criterion is met if both of the required phenotypes are present.^1^ | |

^1^ The phenotypes were required to be in the same segment.

EPD, endplate damage; MC, Modic changes.

2. Data

The MC-size-based criterion demonstrated significant between-group differences in leg pain (p = 0.028). The regression coefficients for the 1/3 group were statistically significant for leg pain (+18.5, p = 0.008) and the Oswestry Disability Index (ODI; +8.3, p = 0.026). Conversely, the positive group did not show statistically significant coefficients for any of the patient-reported outcome measures (PROMs). Additionally, the MEC criterion was associated with significant between-group differences in the EQ-index (p = 0.032); the regression coefficient was statistically significant for the MEC-positive group (−0.069, p = 0.032). Data are provided in Tables 9 and 10.

2.1. MC-size-based criterion

**Table 9** Estimated means of the patient-reported outcome measures (PROMs) and regression coefficients of the groups using the MC-size-based criterion.

|  | PROMs | | | Regression coefficients | | | | | |
| --- | --- | --- | --- | --- | --- | --- | --- | --- | --- |
|  | 0/3 | 1/3 | Positive | Follow-up | | Interaction: 1/3 * follow-up | | Interaction: positive * follow-up | |
|  | Est. mean (s.e.) | Est. mean (s.e.) | Est. mean (s.e.) | B^1^ (s.e.) | p^1^ | B^2^ (s.e.) | p^2^ | B^2^ (s.e.) | p^2^ |
| LBP |  |  |  |  |  |  |  |  |  |
| Baseline | 54.4 (4.5) | 56.6 (2.9) | 52.7 (4.3) |  |  |  |  |  |  |
| Follow-up | 28.7 (4.7) | 30.3 (2.9) | 32.2 (4.7) | -25.7 (5.7) | **<0.001** | -0.64 (6.8) | 0.925 | 5.2 (8.3) | 0.531 |
| p^3^ | 0.696 | | |  |  |  |  |  |  |
| Leg pain |  |  |  |  |  |  |  |  |  |
| Baseline | 70.8 (4.4) | 67.5 (2.8) | 69.0 (4.3) |  |  |  |  |  |  |
| Follow-up | 19.2 (5.0) | 34.5 (3.1) | 29.9 (5.1) | -51.6 (5.9) | **<0.001** | 18.5 (6.9) | **0.008** | 12.5 (8.6) | 0.147 |
| p^3^ | **0.028** | | |  |  |  |  |  |  |
| ODI |  |  |  |  |  |  |  |  |  |
| Baseline | 49.3 (2.2) | 45.4 (1.4) | 47.5 (2.2) |  |  |  |  |  |  |
| Follow-up | 14.1 (2.7) | 18.4 (1.7) | 18.2 (2.7) | -35.3 (3.1) | **<0.001** | 8.3 (3.7) | **0.026** | 6.0 (4.5) | 0.183 |
| p^3^ | 0.085 | | |  |  |  |  |  |  |
| EQ-index |  |  |  |  |  |  |  |  |  |
| Baseline | 0.44 (0.02) | 0.46 (0.01) | 0.44 (0.02) |  |  |  |  |  |  |
| Follow-up | 0.67 (0.03) | 0.66 (0.02) | 0.70 (0.03) | 0.23 (0.03) | **<0.001** | -0.02 (0.04) | 0.609 | 0.04 (0.05) | 0.438 |
| p^3^ | 0.360 | | |  |  |  |  |  |  |
| EQ-VAS |  |  |  |  |  |  |  |  |  |
| Baseline | 40.4 (3.2) | 41.6 (2.1) | 46.9 (3.2) |  |  |  |  |  |  |
| Follow-up | 60.8 (3.8) | 69.4 (2.4) | 75.8 (3.8) | 20.5 (4.5) | **<0.001** | 7.4 (5.4) | 0.170 | 8.4 (6.4) | 0.193 |
| p^3^ | 0.324 | | |  |  |  |  |  |  |

^1^ For the overall effect of the surgery.

^2^ For group-specific interaction (group * follow-up), denoting the relevant group’s effect on the follow-up estimate in relation to the 0/3 reference group.

^3^ For between-group differences in improvement rates.

Adjusted for age, sex, BMI, smoking status, symptom duration, preoperative mental health status, and preoperative motor deficit of leg.

PROMs, patient-reported outcome measures; Est. mean, an estimated mean; s.e., a standard error; B, a regression coefficient; LBP, low back pain; ODI, Oswestry Disability Index.

2.2. Modic-endplate complex

**Table 10** Estimated means of the patient-reported outcome measures (PROMs) and regression coefficients of the groups for the MEC-based analysis.

|  | PROMs | | Regression coefficients | | | |
| --- | --- | --- | --- | --- | --- | --- |
|  | MEC-negative | MEC-positive | Follow-up | | Interaction: MEC-positive * follow-up | |
|  | Est. mean (s.e.) | Est. mean (s.e.) | B^1^ (s.e.) | p^1^ | B^2^ (s.e.) | p^2^ |
| LBP |  |  |  |  |  |  |
| Baseline | 58.4 (3.2) | 52.8 (3.0) |  |  |  |  |
| Follow-up | 30.8 (3.3) | 30.3 (3.1) | -22.5 (3.7) | **<0.001** | -5.1 (5.4) | 0.344 |
| p^3^ | 0.344 | |  |  |  |  |
| Leg pain |  |  |  |  |  |  |
| Baseline | 70.8 (3.1) | 67.4 (3.0) |  |  |  |  |
| Follow-up | 30.8 (3.5) | 30.5 (3.4) | -36.9 (3.9) | **<0.001** | -3.0 (5.6) | 0.587 |
| p^3^ | 0.587 | |  |  |  |  |
| ODI |  |  |  |  |  |  |
| Baseline | 47.9 (1.6) | 45.8 (1.5) |  |  |  |  |
| Follow-up | 17.7 (1.9) | 17.0 (1.8) | -28.7 (2.1) | **<0.001** | -1.5 (3.0) | 0.625 |
| p^3^ | 0.625 | |  |  |  |  |
| EQ-index |  |  |  |  |  |  |
| Baseline | 0.47 (0.01) | 0.43 (0.01) |  |  |  |  |
| Follow-up | 0.65 (0.02) | 0.69 (0.02) | 0.26 (0.02) | **<0.001** | -0.069 (0.03) | **0.032** |
| p^3^ | **0.032** | |  |  |  |  |
| EQ-VAS |  |  |  |  |  |  |
| Baseline | 40.0 (2.3) | 44.7 (2.2) |  |  |  |  |
| Follow-up | 65.4 (2.7) | 40.0 (2.3) | 27.6 (3.0) | **<0.001** | -2.2 (4.3) | 0.611 |
| p^3^ | 0.611 | |  |  |  |  |

^1^ For the overall effect of the surgery.

^2^ For group-specific interaction (group * follow-up), denoting the relevant group’s effect on the follow-up estimate in relation to the MEC-negative reference group.

^3^ For between-group differences in improvement rates.

Adjusted for age, sex, BMI, smoking status, symptom duration, preoperative mental health status, and preoperative motor deficit of leg.

PROMs, patient-reported outcome measures; MEC, the Modic–endplate complex; Est. mean, an estimated mean; s.e., a standard error; B, a regression coefficient; LBP, low back pain; ODI, Oswestry Disability Index.

References

[1] L. Chen, X. Hu, J. Zhang, M.C. Battié, X. Lin, Y. Wang, Modic Changes in the Lumbar Spine are Common Aging-related Degenerative Findings that Parallel With Disk Degeneration, Clinical Spine Surgery: A Spine Publication 31 (2018) 312–317. https://doi.org/10.1097/BSD.0000000000000662.

[2] N.A. Farshad-Amacker, A. Hughes, R.J. Herzog, B. Seifert, M. Farshad, The intervertebral disc, the endplates and the vertebral bone marrow as a unit in the process of degeneration, Eur Radiol 27 (2017) 2507–2520. https://doi.org/10.1007/s00330-016-4584-z.

[3] J.H. Määttä, J. Karppinen, M. Paananen, C. Bow, K.D.K. Luk, K.M.C. Cheung, D. Samartzis, Refined Phenotyping of Modic Changes, Medicine 95 (2016) e3495. https://doi.org/10.1097/MD.0000000000003495.

[4] J. Saukkonen, J. Määttä, P. Oura, E. Kyllönen, O. Tervonen, J. Niinimäki, J. Auvinen, J. Karppinen, Association Between Modic Changes and Low Back Pain in Middle Age, Spine (Phila Pa 1976) 45 (2020) 1360–1367. https://doi.org/10.1097/BRS.0000000000003529.

[5] J.D. Baker, A.J. Sayari, G.K. Harada, Y. Tao, P.K. Louie, B.A. Basques, F. Galbusera, F. Niemeyer, H. Wilke, H.S. An, D. Samartzis, The Modic‐endplate‐complex phenotype in cervical spine patients: Association with symptoms and outcomes, Journal of Orthopaedic Research 40 (2022) 449–459. https://doi.org/10.1002/jor.25042.
